# Metabolic dynamic score and machine learning: a novel approach to predicting pathological complete response in rectal cancer after neoadjuvant chemoradiotherapy

**DOI:** 10.3389/fonc.2026.1910909

**Published:** 2026-08-07

**Authors:** Tengyi Peng, Qiqi Zhang, Xingrong Lai, Zhen Pan, Bin Chen, Zhicheng Zhuang, Shaoqing Zheng, Xing Liu, Jinfu Zhuang, XingRong Lu, Guoxian Guan, Shoufeng Li

**Affiliations:** 1 Department of Colorectal Surgery, The First Affiliated Hospital of Fujian Medical University, Fuzhou, China; 2 Department of Internal Medicine, Fujian Qingliu County General Hospital, Sanming, China; 3 Department of Colorectal Surgery, National Regional Medical Center, Binhai Campus of the First Afliated Hospital, Fuzhou, China; 4 Department of Colorectal Surgery, Fujian Medical University Union Hospital, Fuzhou, China

**Keywords:** biochemical indicators, locally advanced rectal cancer, machine learning model, neoadjuvant chemoradiotherapy, pathological complete response

## Abstract

**Background:**

This study developed a scoring system based on the dynamic changes in biochemical indicators in advance of and subsequent to neoadjuvant chemoradiotherapy (NCRT) in individuals with locally advanced rectal cancer (LARC). The scoring system, combined with other clinical features, was used to develop a machine learning (ML) model aimed at predicting a pathological complete response (pCR).

**Methods:**

A review of earlier data was performed on the data of 1300 patients with LARC treated at Center1 and Center 2. To determine factors linked to pCR and create the scoring system, uni and multivariate logistic regression analyses were conducted. The development of predictive models involved the use of 10 ML methods, while model performance was evaluated using the area under the receiver operating characteristic curve (AUC), area under the precision-recall curve (AUPRC), decision curve analysis, and calibration curves. Additionally, SHapley Additive exPlanations values were applied to enhance model interpretability.

**Results:**

Multivariate analysis identified the MDS, tumor size, clinical T stage, and clinical N stage as independent predictors, which were incorporated into the ML models. Among the 10 ML models, the XGBoost model demonstrated the best and most generalizable predictive performance (training set: AUC = 0.93, AUPRC = 0.732; external validation set: AUC = 0.92, AUPRC = 0.779) and was selected as the optimal model.

**Conclusion:**

This study analyzed changes in biochemical indicators pre- versus post-NCRT and developed a promising MDS. By leveraging ML predictive models to estimate pCR, this approach may serve as a convenient tool to support the clinical decision-making process pending further prospective validation.

## 1 Introduction

Colorectal cancer ranks among the most common cancer types globally, comprising 3.2% of cancer-related deaths (1). Among newly diagnosed rectal cancer cases annually, 30% are locally advanced with poor survival rates (2). The usual approach for treating locally advanced rectal cancer (LARC) includes neoadjuvant chemoradiotherapy (NCRT) and surgery (1, 3). Better clinical outcomes are often observed in patients who obtain a pathological complete response (pCR) after NCRT (4, 5). Therefore, the accurate prediction of pCR is critical for clinical decision-making in LARC management.

The significance of serum metabolic indicators in cancer biology has gained increasing recognition in recent years (6–8). Rectal cancer is a malignant tumor closely associated with metabolic disorders, particularly those involving glucose and lipid metabolism (9–11). Glucose metabolism promotes the initiation and progression of rectal cancer by activating proto-oncogenes, inactivating tumor suppressor genes, inducing abnormal expression of metabolic enzymes, and activating various signaling pathways—all of which facilitate macromolecule synthesis and massive adenosine triphosphate (ATP) production to fuel tumor growth (12). Meanwhile, lipid metabolism drives rectal cancer progression by producing ATP through upstream factor acetylation, cell death modulation, and the activation of fatty acid β-oxidation–regulating pathways (13, 14). Lipid and glucose metabolism are closely interrelated, synergistically meeting the heightened energy demands of cancer cells (15). Therefore, both glucose and lipid metabolism play important roles in the occurrence and development of rectal cancer. The blood biochemistry index can partially indicate changes in organism metabolism. Blood biochemical indicators have practical advantages in predicting pCR due to their universal examination and easy availability. However, previous studies have evaluated only the predictive value of metabolic markers either pre- or post-NCRT (16, 17), neglecting the dynamic changes in these markers across the treatment course.In addition, functional imaging modalities such as PET/CT have also shown promise in predicting pCR, particularly through baseline metabolic parameters like metabolic tumor volume and total lesion glycolysis (18).

Machine learning (ML) has been widely adopted in predictive modeling. Compared to traditional statistical models, ML offers superior adaptability to nonlinear relationships and enhanced modeling capabilities (19–21). In rectal cancer management, ML has demonstrated utility in predicting postoperative complications, distant metastasis, and recurrence (22–24). While prior research has employed ML to predict pCR in patients with LARC prior to treatment (25), no studies have leveraged dynamic changes in metabolic markers to predict pCR after NCRT.

Here, we developed a novel predictive metric, the Metabolic Dynamic Score (MDS), which quantifies the integrated dynamic changes of key serum metabolic markers before and after NCRT. This metric was integrated with clinicopathological features to construct ML models using 10 distinct algorithms, aiming to predict post-NCRT pCR. The MDS is designed to capture the systemic metabolic response to therapy, providing a composite biomarker that may reflect the perturbation and potential restoration of host-tumor metabolic homeostasis. Our model shows potential to assist clinicians in therapeutic decision-making, though further validation is required before routine application.

## 2 Materials and methods

### 2.1 Study population

This retrospective study analyzed clinicopathological data from 1300 patients with LARC treated at two medical centers - 846 at Center 1 and 454 at Center 2– between June 2010 and July 2017. The initial clinical staging for all patients was based on high-resolution pelvic magnetic resonance imaging (MRI). To rule out distant metastasis, some patients also underwent chest/abdominal/pelvic CT scans.​ All patients were managed within our institution’s routine multidisciplinary team (MDT) framework. Treatment decisions were made following discussion by a fixed MDT comprising experts from gastrointestinal surgery, medical oncology, radiation oncology, radiology, pathology, and clinical nutrition.

All enrolled patients received preoperative neoadjuvant chemoradiotherapy. The radiotherapy was delivered using intensity-modulated radiotherapy (IMRT) technique. The clinical target volume included the primary tumor, mesorectum, and regional lymph nodes. The radiotherapy protocol involved a total pelvic dose of 45 Gy delivered in 25 fractions (five fractions/week) over 5 weeks with an additional 5.4-Gy boost to the primary tumor. Concurrent chemotherapy regimens included CapeOX (capecitabine 1000 mg/m^2^ orally twice daily on days 1–14 plus oxaliplatin 130 mg/m^2^ intravenously on day 1, every 3 weeks), capecitabine as a single agent (825 mg/m^2^ orally twice daily throughout radiotherapy), and FOLFOX (5-fluorouracil plus oxaliplatin according to standard dosing).Given the limited sample size within each chemotherapy subgroup and the retrospective nature of this study, we did not perform formal subgroup comparisons across different regimens.

Surgery was performed 6–8 weeks after completion of NCRT. The surgical approach was determined by tumor location: total mesorectal excision (TME) for tumors located in the mid- or lower rectum and partial TME with 5-cm distal margin preservation for high rectal cancers (26). All patients received adjuvant chemotherapy 4–8 weeks postoperatively, regardless of the surgical pathology findings. The following stringent exclusion criteria were applied: synchronous distant metastasis at surgery, survival < 1 month, or lacking clinical/pathological data. Approval for the study protocol was granted by the review boards of the two participating hospitals.

### 2.2 Data collection

Patient data gathered from electronic health records included pre- and post-NCRT biochemical parameters (total bilirubin, direct bilirubin, indirect bilirubin, gamma-glutamyl transferase [GGT], alkaline phosphatase [ALP], triglycerides [TG], total cholesterol, high-density lipoprotein [HDL], low-density lipoprotein [LDL], apolipoprotein A, apolipoprotein B, glucose, and lactate dehydrogenase [LDH]), clinical T/N stage, tumor size, presence of lymphovascular or perineural invasion, distance from the anal verge, and pCR status. A scoring system was subsequently developed based on uni and multivariate analyses of these metabolic indicators.

### 2.3 Construction of the metabolic dynamic score

The MDS is a composite variable designed to integrate predictive information from key serum metabolic markers measured at two critical clinical time points: before and after NCRT. From the complete panel of biochemical parameters, the raw values of all indicators at both pre- and post-NCRT time points and their changes (Δ) were initially considered. Variables showing a significant association with pCR (P < 0.05) in a univariate logistic regression analysis, conducted strictly on the training set, were selected as candidates.To ensure comparability, all continuous candidate variables underwent Z-score standardization using parameters (mean and standard deviation) calculated solely from the training set; these same parameters were then applied to transform the corresponding variables in the validation sets to prevent data leakage. These standardized variables were entered into a multivariable logistic regression model, again fitted exclusively on the training set, to identify independent predictors. The final MDS is defined as the linear predictor (i.e., the weighted sum) of this fitted multivariable model: MDS = Σ (β_i * Z_i) + Intercept, where Z_i is the standardized value of the i-th retained independent variable and β_i is its corresponding regression coefficient. This approach consolidates information from multiple markers at distinct time points into a single metric. The specific variables constituting the MDS and their regression coefficients are detailed in **Table 1**. Clinical T and N staging followed the American Joint Committee on Cancer’s 8th edition of the Tumor Node Metastasis classification system. Tumor regression grade was assessed based on pathological response to chemoradiotherapy using the same criteria. A pCR is defined as the presence of no viable tumor cells in the primary tumor and regional lymph nodes.

**Table 1:** Univariate and multivariate logistic regression analyses of MDS associated with pCR.

Indicators	Univariate analysis			Multivariate analysis		
	β	HR(95%CI)	P-value	β	HR(95%CI)	P-value
Pre-GGT	-0.027	0.973(0.960-0.986)	**<0.001**	-0.020	0.980(0.967-0.994)	**0.004**
Pre-ALP	-0.020	6.219(3.462-11.172)	**<0.001**	-0.014	0.98(0.975-0.997)	**0.013**
Δ-LDL	1.828	1.162(1.045-1.292)	**<0.001**	0.908	1.085(0.970-1.214)	**0.014**
Post-HDL	1.907	6.732(3.777-11.998)	**<0.001**	0.906	2.473(1.205-5.076)	**0.020**
Pre-APO-A	1.900	6.688(3.371-13.269)	**<0.001**	1.034	2.811(1.206-6.553)	**0.017**

*MDS = [(-0.020× GGT) + (-0.014 × ALP) + (0.908×Δ-LDL) + (0.906×Post-HDL) + (1.034×APO-A)].

(GGT, Gamma Glutamyl Transferase; ALP, Alkaline phosphatase; LDL, Low-density lipoprotein; HDL, High-density lipoprotein; APO-A, apolipoprotein A; pCR, pathological complete response).

Values in bold type indicate that the p-value is less than 0.05, which is considered statistically significant.

### 2.4 Feature selection and model development

To prevent data leakage, all feature selection procedures were strictly confined to the training set. First, univariate logistic regression was performed on the training set to identify variables with P < 0.05 for potential association with pathological complete response (pCR). These selected variables were then entered into a multivariable logistic regression analysis, also exclusively on the training set, to identify independent predictors. The final set of variables retained from this process—namely, the MDS (constructed from the independent predictors), tumor size, cT, and cN—was subsequently used for all further model development and evaluation.

For predictive modeling, we employed ten machine learning algorithms: extreme gradient boosting (XGBoost), random forest, decision tree, logistic regression, light gradient boosting machine, adaptive boosting, k-nearest neighbors, support vector machine, naïve Bayes classifier, and multilayer perceptron. The 846 patients from Center 1 were randomly split into a training set (70%, *n* = 650) and an internal validation set (30%, *n* = 196) using a stratified split based on pCR status to preserve the outcome distribution. The 454 patients from Center 2 served as a completely independent external validation set.

Hyperparameter tuning was performed via a random search combined with 10-fold cross-validation (stratified by pCR status to ensure consistent class proportions across folds), with the optimal hyperparameters selected based on the highest average cross-validated AUC. For the XGBoost model, we specifically searched over the following ranges: n_estimators = 1000, max_depth = 3. Additionally, early stopping with a patience of 50 rounds on the validation set was applied during XGBoost training to mitigate overfitting.

To further ensure generalizability, we implemented multiple strategies against overfitting: (1) internal validation on the held-out Center 1 set, (2) external validation on the fully independent Center 2 cohort, (3) 10-fold cross-validation during training, and (4) early stopping for the XGBoost model.

Model performance was evaluated using the area under the receiver operating characteristic curve (AUC) and the area under the precision-recall curve (AUPRC)—the latter being particularly informative for the imbalanced pCR outcome. Decision curve analysis and calibration curves were also employed to assess clinical utility and reliability. For the best-performing model, the SHapley Additive exPlanations (SHAP) framework was used to quantify the contribution of each feature.

### 2.5 Statistical analysis

SPSS Statistics (version 23) was employed for the statistical analysis, while ML models were implemented in Python (version 3.8). Categorical variables are presented as numbers (percentages) and compared using the chi-square or Fisher’s exact test. Continuous variables are presented as mean ± standard deviation or median (interquartile range) and compared using the Student’s t-test or Mann-Whitney U test, as appropriate. To identify the independent predictors for the MDS and the subsequent nomogram, only variables with values of P<0.05 on the univariate analysis were considered in the multivariate analysis. Based on the final multivariable logistic regression model, a nomogram was constructed to visualize the prediction of pCR probability. To evaluate the model’s performance, AUC, AUPRC, calibration curves for both the ML models and the nomogram, and decision curve analysis were employed.

### 2.6 Model reproducibility, code availability, and bias sensitivity analysis

This work has been reported in accordance with the STROCSS criteria (27). To ensure full reproducibility, the complete Python code used for data preprocessing, Metabolic Dynamic Score calculation, feature selection, machine learning model training, hyperparameter tuning, evaluation, and SHAP analysis has been made publicly available on GitHub (https://github.com/panzhen1998/MDS.git).

## 3 Results

### 3.1 Patient clinical characteristics

**Figure 1:**
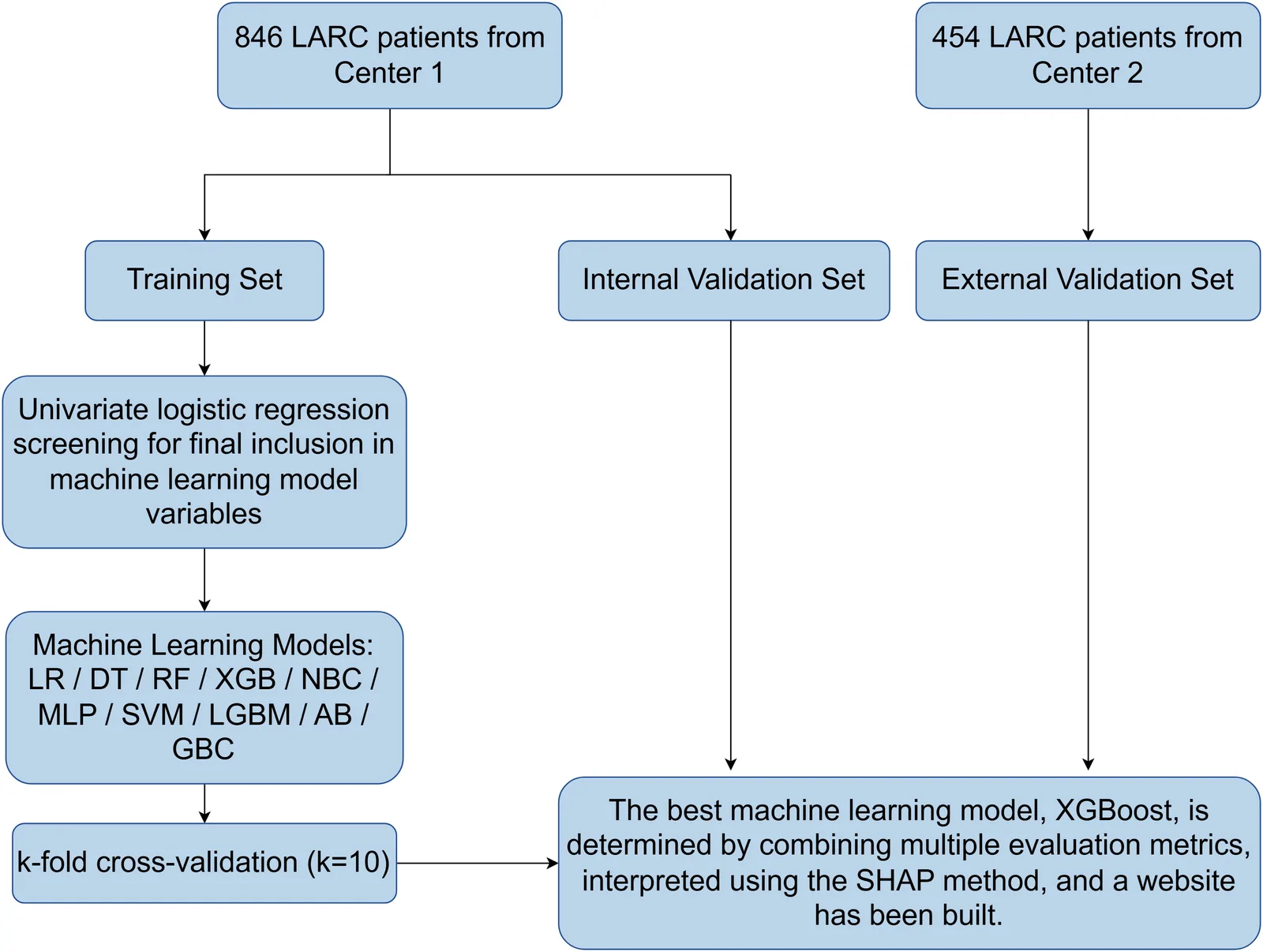
Flowchart for the establishment of MDS and machine learning prediction models.

Our study comprised 1300 patients with LARC from the Center 2 (n = 454) and Center 1 (n = 846). Patients from Center 1 were randomly split into a training set (70%, n=650) and an internal validation set (30%, n=196) using a stratified split based on pCR status. Patients from Center 2 served as a completely independent external validation set (n=454). The patient selection process is detailed in **Figure 1**. **Table 2** displays the clinical features of patients across the training, internal validation, and external validation sets. The detailed distributions of all candidate metabolic indicators—including pre-NCRT levels, post-NCRT levels, and their changes (Δ)—for these cohorts are provided in **Supplementary Table 1**. No statistically significant intergroup differences were observed in the baseline clinical characteristics or in the pre- and post-treatment metabolic indicators among the three sets.

**Table 2:** Baseline characteristics.

Variables	Center 1		Center 2 N = 454	P
	Training set N = 650	Internal validation set N = 196		
Age	57.50 ± 11.20	56.60 ± 10.75	57.89 ± 11.37	0.178
Gender, n(%)				0.842
Female	299(46.00)	89(45.41)	210 (46.26)	
Male	351 (54.00)	107 (54.59)	244 (53.74)	
Postoperative hospital stay	8.69 ± 5.30	8.36 ± 5.40	8.83 ± 5.25	0.299
Interval between neoadjuvant chemoradiation and surgery	63.15 ± 11.08	62.09 ± 11.30	63.61 ± 10.97	0.110
ASA class, n(%)				
1	489 (75.23)	138 (70.41)	351 (77.31)	0.091
2	151 (23.23)	56 (28.57)	95 (20.93)	
3	10 (1.54)	2 (1.02)	8(1.76)	
cT, n(%)				0.063
cT_1_	4 (0.62)	3 (1.53)	1 (0.22)	
cT_2_	30 (4.62)	8 (4.08)	22 (4.85	
cT_3_	250 (38.46)	65 (33.16)	185 (40.74)	
cT_4_	366 (56.31)	120 (61.22)	246 (54.19)	
cN, n(%)				0.279
cN_0_	151 (23.23)	54 (27.55)	97 (21.37)	
cN_+_	499 (76.77)	142 (72.45)	357 (78.63)	
Distance from anal verge (cm)	5.65 ± 2.01	5.52 ± 1.95	5.70 ± 2.03	0.292
Tumor diameter	2.77 ± 1.32	2.91 ± 1.36	2.70 ± 1.31	0.063
pCR, n(%)				0.640
No	505 (77.69)	150 (76.53)	355 (78.19)	
Yes	145 (22.31)	46 (23.47)	99 (21.81)	
Metabolism score x:High>0.398, Low<0.398, n(%)				0.542
High	373 (57.38)	116 (59.18)	257 (56.61)	
Low	277 (42.62)	80 (40.82)	197 (43.39)	
Chemotherapy and radiotherapy side effects, n(%)				0.184
No	493 (75.85)	142 (72.45)	351 (77.31)	
Yes	157 (24.15)	54 (27.55)	103 (22.69)	
Nerve invasion, n(%)				0.908
No	562 (86.46)	169 (86.22)	393 (86.56)	
Yes	88 (13.54)	27 (13.78)	61 (13.44)	
Lymphovascular invasion, n(%)				0.765
No	628 (96.62)	190 (96.94)	438 (96.48)	
Yes	22 (3.38)	6 (3.06)	16 (3.52)	
Postoperative complications, n(%)				0.963
No	528 (81.23)	159 (81.12)	369 (81.28)	
Yes	122 (18.77)	37 (18.88)	85 (18.72)	

ASA, American Society of Anesthesiologists.

### 3.2 Establishment of MDS

Here, we analyzed all metabolic indicators of the enrolled patients with LARC pre- and post-NCRT. Using logistic regression analysis, we identified that pre-NCRT GGT, pre-NCRT ALP, Δ-LDL, post-NCRT HDL, and apolipoprotein A were correlated with pCR (**Table 1**). By multiplying the logistic coefficients with the respective indicators, the MDS was derived as follows: Score = -0.020GGT - 0.014×ALP - 0.908×Δ-LDL + 0.906×post-NCRT HDL + 1.034×Apolipoprotein A (**Table 1**). The optimal cutoff value of 0.398 was determined using the ROC curve for the derived score of 0.779, categorizing patients into high- and low-score groups.

### 3.3 Uni and multivariate logistic regression analysis

The training set data underwent uni and multivariate logistic regression analyses of MDS, age, tumor distance from the anal verge, tumor size, sex, clinical T stage (cT), clinical N stage (cN), lymphovascular invasion, perineural invasion, radiotherapy complications, and postoperative complications. **Table 3** displays the uni and multivariate logistic regression analyses results, which revealed that tumor size, MDS, cT, and cN (P<0.05) were distinct risk factors for patients with LARC. These independent risk factors were subsequently integrated into the ML predictive model.

**Table 3:** Logistic univariate and multivariate analysis of predictors for pCR.

Variable	Univariate analysis		Multivariate analysis	
	OR (95%CI)	P-value	OR (95%CI)	P-value
Mean, age (year)	1.000(0.984-1.017)	0.962		
Mean, distance from the anal verge(cm)	0.992(0.905-1.088)	0.865		
Mean, tumor size(cm)	0.621(0.519-0.744)	**<0.001**	0.810(0.656-0.963)	**0.050**
Adjuvant chemotherapy		0.925		
No	1			
Yes	0.981(0.662-1.455)			
Sex		0.417		
Female	1			
Male	0.858(0.593-1.242)			
Metabolism score		**<0.001**		**<0.001**
Low	1		1	
High	2.890(1.972-4.235)	**<0.001**	2.193(1.220-3.942)	**<0.001**
Clinical T stage		**<0.001**		**<0.001**
cT_1_	1		1	
cT_2_	1.095(0.098-12.296)	0.941	0.132(0.007-2.570)	0.181
cT_3_	0.108(0.011-1.053)	0.055	0.024(0.001-0.443)	**0.012**
cT_4_	0.063(0.006-0.614)	**0.017**	0.012(0.001-0.220)	**0.003**
Clinical N stage		**<0.001**		**<0.001**
cN_0_	1		1	
cN_+_	0.015(0.008-0.026)		0.029(0.009-0.101)	
Lymphovascular invasion		0.511		
No	1			
Yes	1.257(0.635-2.488)			
Neural invasion		0.062		
No	1			
Yes	0.440(0.185-1.044)			
Radiotherapy complication		0.274		
No	1			
Yes	1.263(0.831-1.920)			
Postoperative complication		0.300		
No	1			
Yes	1.285(0.800-2.063)			

LARC, locally advanced rectal cancer; pCR, pathological complete response; SD, Standard deviation; HR, Hazard ratio; CI, Confidence interval.

Values in bold type indicate that the p-value is less than 0.05, which is considered statistically significant.

### 3.4 Construction and validation of a predictive nomogram

**Figure 2:**
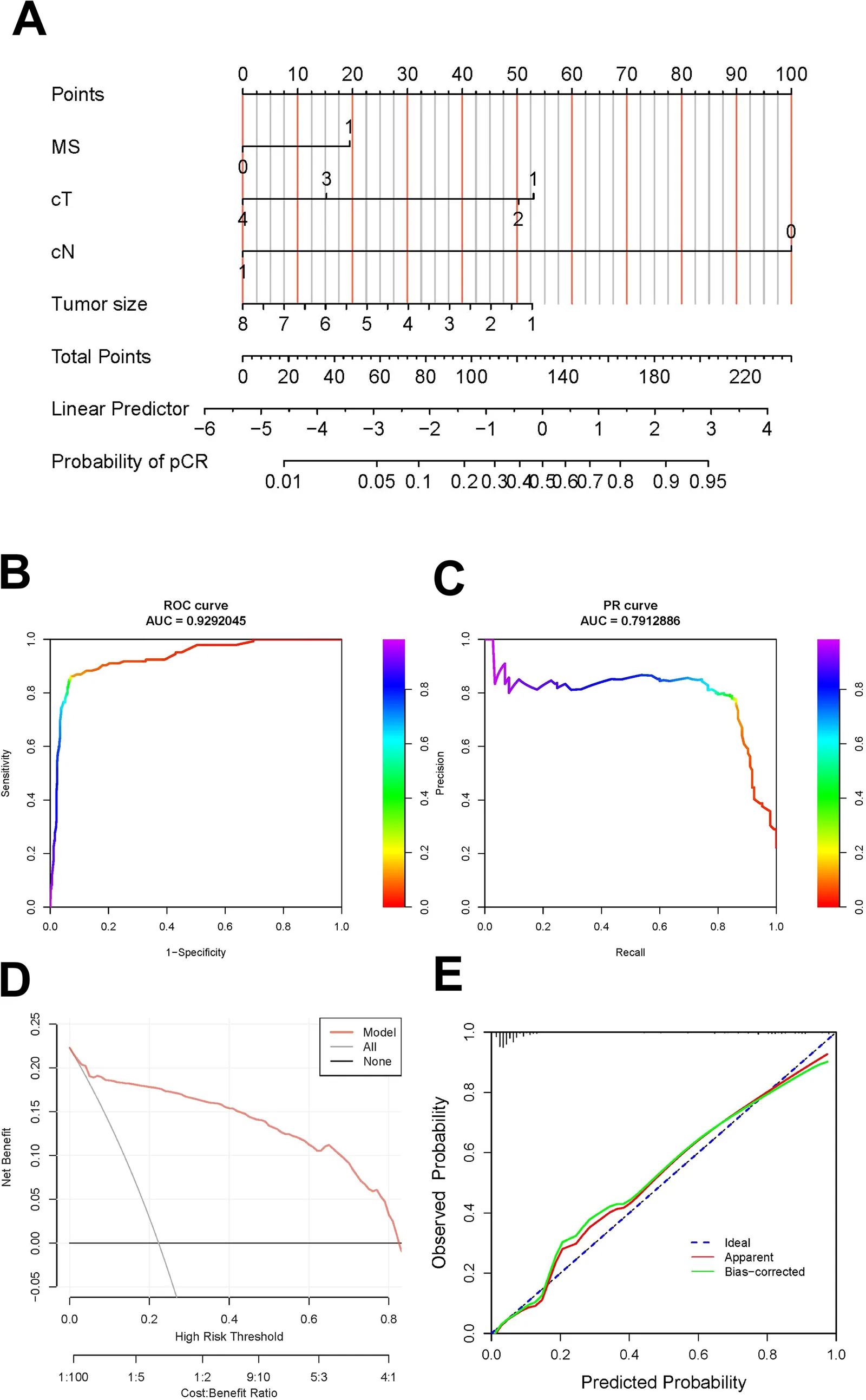
Development and validation of a nomogram for predicting pathological complete response (pCR). Nomogram: Assigns scores to four independent predictors (MDS, clinical T stage, clinical N stage, tumor size) based on their values. Summing the scores of these predictors yields a total score, which can be mapped to the bottom scale to estimate an individual patient’s probability of pCR **(A)**. ROC curve: Evaluates the nomogram’s ability to distinguish between patients with and without pCR; the color gradient indicates varying risk thresholds **(B)**. Precision-recall (PR) curve: Assesses the nomogram’s performance for the imbalanced pCR outcome; the color gradient corresponds to risk thresholds **(C)**. Decision curve analysis: Compares the clinical net benefit of the nomogram against “treat all” and “treat none” strategies across different conditions **(D)**. Calibration curve: Validates the agreement between the nomogram’s predicted pCR probabilities and observed pCR frequencies; the dashed line represents ideal calibration, the red line denotes apparent calibration, and the green line indicates bias-corrected calibration **(E)**.

To transform the statistical model into a clinically usable tool, a predictive nomogram was developed based on the four independent predictors identified in the multivariate logistic regression analysis (**Table 3**): the MDS, clinical T stage, clinical N stage, and tumor size (**Figure 2A**). Each predictor is assigned a score on the “Points” scale. By summing the individual scores corresponding to a patient’s specific values, a “Total Points” can be obtained, which is then projected to the bottom scale to estimate the individualized probability of achieving pCR. The performance of this nomogram was rigorously evaluated. It demonstrated excellent discriminative ability, with an AUC of 0.929 (95% CI: 0.902–0.956) (**Figure 2B**). The precision-recall curve, suitable for imbalanced outcomes, yielded an average precision (AP) of 0.791 (**Figure 2C**). Decision curve analysis confirmed the clinical net benefit of using the nomogram for pCR prediction across a wide range of threshold probabilities (**Figure 2D**). Furthermore, the calibration curve showed strong agreement between the nomogram-predicted probabilities of pCR and the actual observed frequencies, with the bias-corrected curve closely aligning with the ideal reference line (**Figure 2E**). These results validate the nomogram as a robust and clinically applicable predictive instrument.

### 3.5 Performance of predictive model

**Figure 3:**
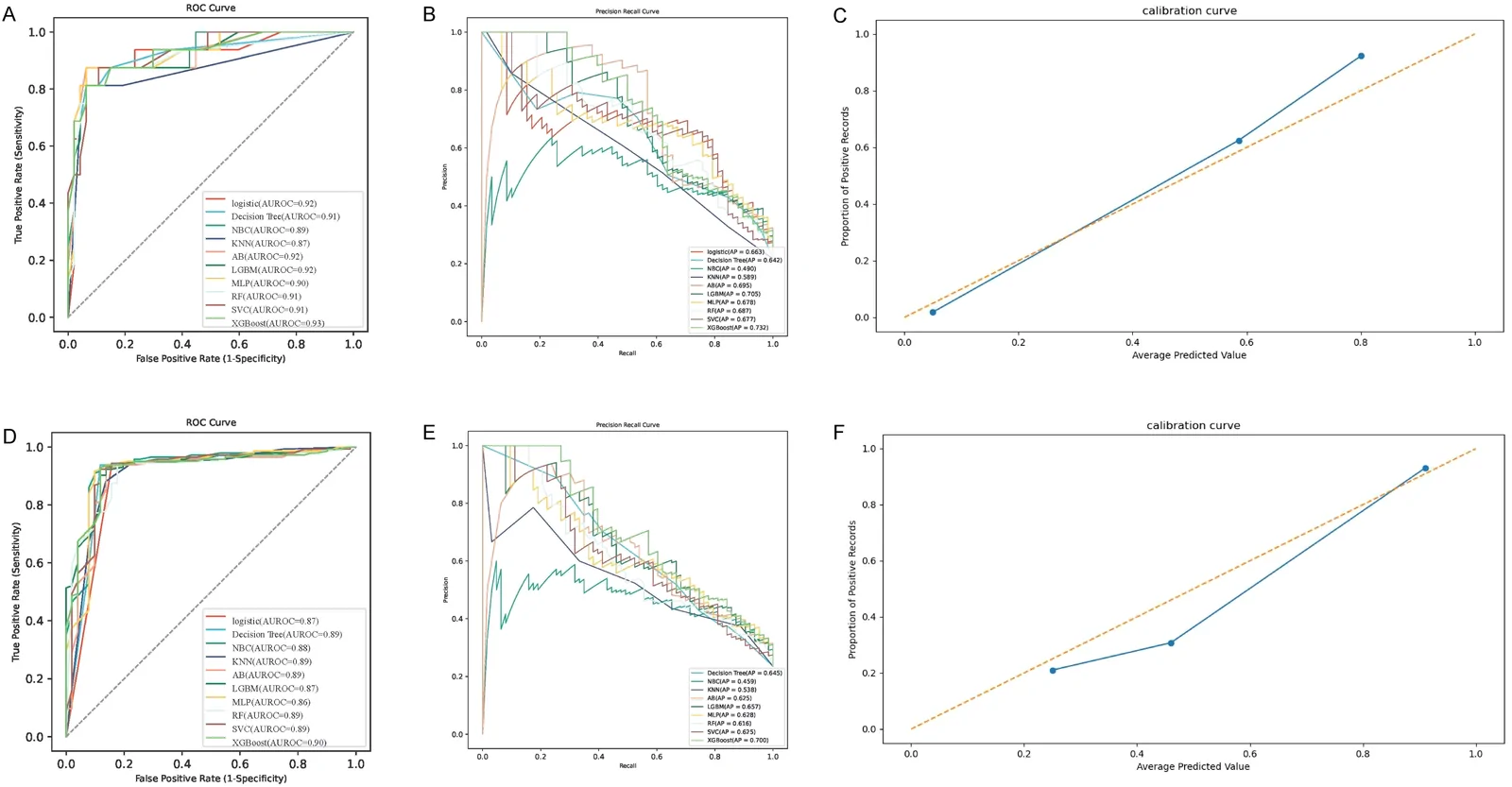
ROC curves of ten machine learning models in the training set **(A)**, PR curves of ten machine learning models in the training set **(B)**, Calibration curves of XGB machine learning model in the training set **(C)**, ROC curves of ten machine learning models in the Internal validation set **(D)**, PR curves of ten machine learning models in the Internal validation set **(E)**, Calibration curves of XGB machine learning model in the Internal validation set **(F)**.

**Figure 4:**
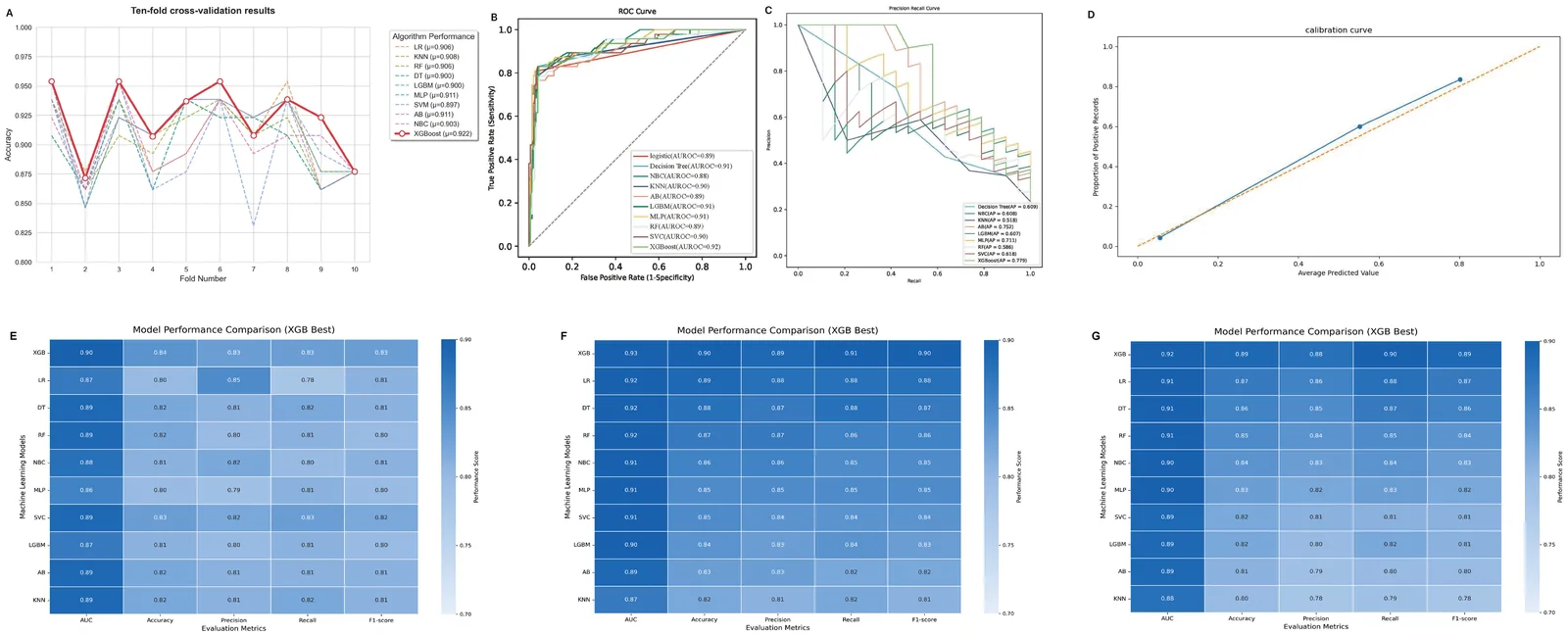
Ten-fold cross-validation results of ten machine models in the training set **(A)**, ROC curves of ten machine learning models in the External validation set **(B)**, PR curves of ten machine learning models in the External validation set **(C)**, Calibration curves of XGB machine learning model in the External validation set **(D)**,Heatmap of the XGBoost model’s performance on the training set **(E)**,Heatmap of the XGBoost model’s performance on the Internal validation set **(F)**,Heatmap of the XGBoost model’s performance on the External validation set **(G)**.

The training dataset was employed to construct predictive models using 10 different ML techniques. Among them, the XGBoost model demonstrated superior and consistent performance, achieving the highest average AUC of 0.93 and AP of 0.732 in the training set (**Figures 3A, B**). Its robust performance was confirmed through 10-fold cross-validation (**Figure 4A**) and in the internal validation set (**Figures 3D, E**). The model’s generalizability was further validated on the completely independent external validation set, where it attained the highest AUC (0.92) and AP (0.779) (**Figures 4B, C**), with heatmap comparisons (**Figures 4E–G**) confirming its advantage over other algorithms. To assess potential algorithmic bias, a fairness evaluation was conducted by analyzing the XGBoost model’s performance across gender subgroups in the external validation set. The model showed excellent and comparable discrimination in both male (AUC = 0.92) and female (AUC = 0.91) subgroups, with no statistically significant difference per DeLong’s test (P = 0.76), indicating no significant performance bias related to gender in our cohort; detailed results are provided in **Supplementary Table 2**. Based on this comprehensive evaluation, the XGBoost model was selected as the optimal predictive tool. To facilitate clinical application, an interactive web-based tool was developed, accessible via a provided stable link, with an interface screenshot available in **Supplementary Figure 1**.

### 3.6 SHAP value interpretation

**Figure 5:**
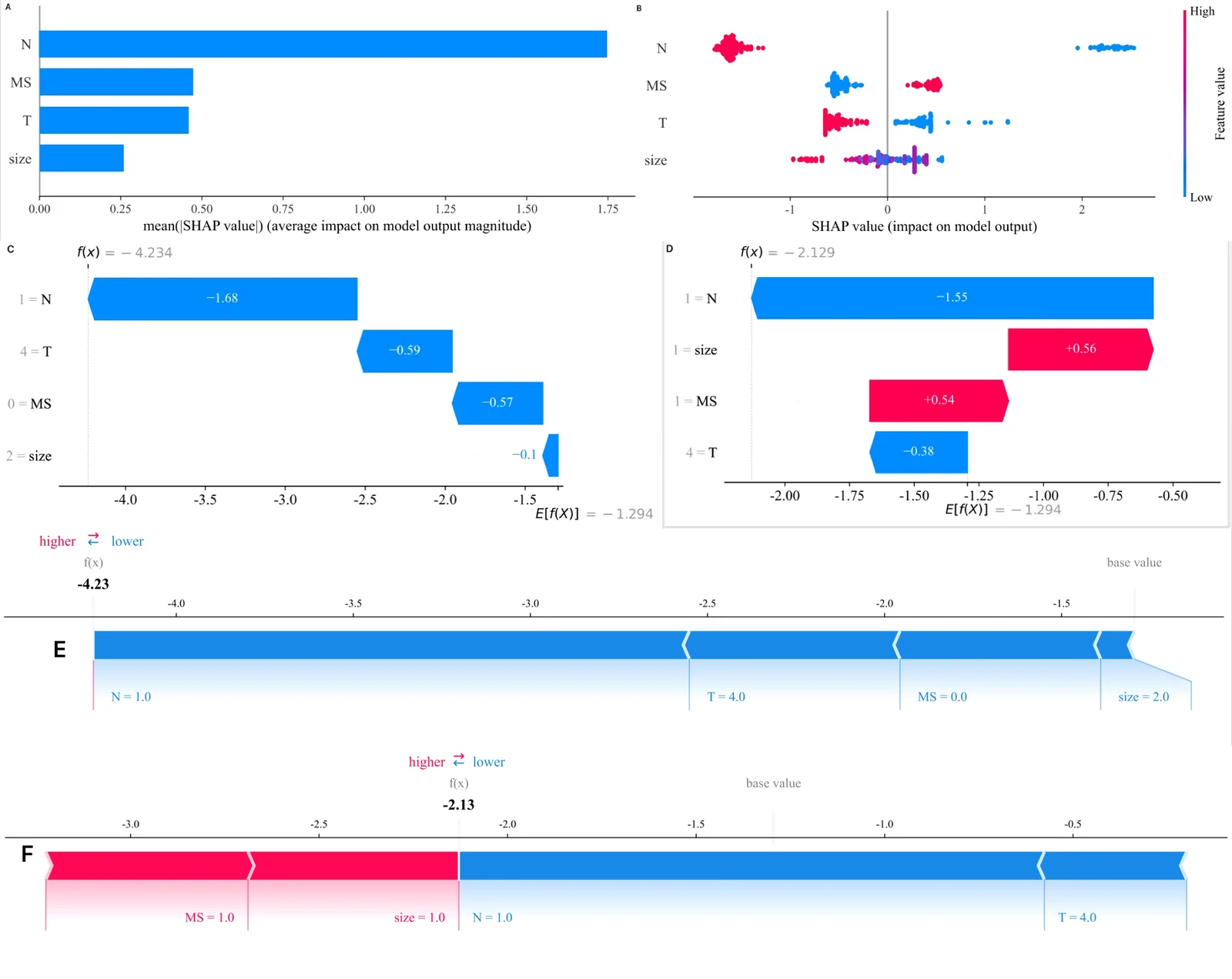
Relative importance of variables based on SHAP for XGB prediction model **(A)** bar chart showing the mean SHAP values; **(B)** SHAP summary plot; **(C, D)** SHAP dependence plots for different features; **(E, F)** SHAP force/decision plots). SHAP, Shapley's Additive explanations; XGB, extreme gradient boosting; Metabolic Dynamic Score (MDS); clinical N-stage (N); clinical T-stage (T); and tumor size. Predictive performance of 10 models in the training set, internal test set, external validation set. Extreme gradient boosting (XGB), random forest (RF), decision tree (DT), logistic regression (LR), light gradient boosting (LGBM), adaptive boosting (AB), K-nearest neighbor (KNN), support vector machine (SVM), naive Bayes (NBC), and multilayer perceptron (MLP).

To enhance the interpretability of the predictive model using ML, the impact of each variable on the outcome was visualized using SHAP values. **Figure 5A** highlights the relative importance of the variables, with cN having the greatest impact, followed by MDS, cT, and tumor size. Regarding variable correlations, **Figure 5B** shows that cN, cT, and tumor size were negatively correlated with the outcome, whereas MDS was positively correlated. **Figures 5C, D** display the SHAP values for specific samples, illustrating how each feature (cN, MDS, cT, tumor size) contributes to the deviation of the model’s prediction from the baseline value (E[f(X)]). Blue (or negative) typically indicate a reduction in predicted values, while red (or positive) indicates an increase. **Figures 5E, F** illustrate the combined effects of different feature value combinations on model output, with color gradients (e.g., blue to red) reflecting changes in model output as feature values shift from lower to higher.

## 4 Discussion

Here we formulated a novel scoring system based on dynamic changes in blood metabolic markers pre- versus post-NCRT in patients with LARC. We also employed 10 ML prediction models combining this new MDS predictive factor with clinicopathological features to predict pCR rates in patients with LARC. To determine the most effective predictive model, the effectiveness of these ML models was measured using ROC, precision-recall, and calibration curves. This research utilized relatively accessible metabolic indicators to predict pCR to provide clinicians more individualized and effective decision-making support.

Lipid and glucose metabolism play crucial roles in tumor progression. In recent years, studies have consistently demonstrated a correlation between abnormal glucose metabolism and cancer progression (28, 29), including colorectal cancer (30). Abnormal lipid metabolism in rectal cancer is also closely related to tumor development (31, 32), with research indicating that lipid metabolic pathways have become important therapeutic targets for rectal cancer (33). Given the inseparable relationship between glucose/lipid metabolic pathways and rectal cancer progression, we used metabolic indicators pre- versus post-NCRT to predict pCR. Previous studies employed magnetic resonance imaging and circulating tumor DNA to predict pCR rates in patients with rectal cancer (34, 35); however, these methods are relatively difficult to perform compared to serological indicators. Therefore, we utilized more convenient multiple serological indicators to predict pCR. In this study, we established a metabolic scoring index, the MDS, based on these indicators in patients with LARC receiving NCRT, then combined it with clinical features to predict pCR.

Apolipoprotein A and ALP are important components of lipid metabolism, which is indirectly related to glucose metabolism, and have significant predictive value in rectal cancer progression (36–38). Studies have shown that apolipoprotein A is negatively correlated with NCRT response among patients with LARC (39). Levels of HDL, a key product and participant in lipid metabolism, are positively associated with rectal cancer progression (40). Levels of GGT, an important product in glutathione metabolism, are also correlated with rectal cancer progression (38).

In studies predicting pCR among patients with breast cancer, Chen et al. used metabolic indicators during neoadjuvant therapy to predict pCR. In our study, pre-NCRT GGT, ALP, and Δ-LDL and post-NCRT HDL and apolipoprotein A levels were all significant on the univariate analysis (P<0.05). Previous studies investigated the predictive value of metabolic indicators pre- or post-NCRT (36, 41, 42), but did not examine the dynamic changes of these indicators throughout the NCRT process. However, we developed a new predictive marker, MDS, based on the dynamic changes of these parameters pre- versus post-NCRT. We constructed a new predictive factor, the MDS, based on dynamic changes in these indicators pre- versus post-NCRT, which also showed predictive significance for pCR (P<0.05). The core rationale behind constructing the MDS in this study is that therapy-induced dynamic changes in serum metabolic markers may profoundly reflect the biology of tumor-host interaction and treatment response. The association between a relative increase in post-treatment HDL-C and ApoA-I levels and pCR may be linked to the “lipid trap” phenomenon in rectal cancer. Research indicates that tumor cells, especially under stress, avidly uptake HDL-cholesterol and ApoA-I from the circulation (43) to meet the demands of membrane synthesis and energy metabolism for rapid proliferation, leading to the “trapping” of HDL in the tumor microenvironment and reduced peripheral blood levels. Neoadjuvant chemoradiotherapy, as a potent therapeutic stressor, may initially exacerbate this “trap” effect. However, in treatment-sensitive patients, effective tumor cell kill disrupts this aberrant lipid acquisition program. As the tumor burden decreases, the “stealing” effect on HDL diminishes, potentially leading to a post-treatment “rebound” in peripheral HDL-C and ApoA-I levels (44, 45). Consequently, the dynamic pattern of HDL-C/ApoA-I captured by the MDS, rather than their absolute values at a single time point, may more sensitively indicate the tumor’s metabolic response to therapy and its subsequent regression. Furthermore, beyond lipid transport, HDL-C and ApoA-I possess anti-inflammatory, antioxidant, and immunomodulatory functions (46–48). Their recovery may reflect an improvement in systemic inflammatory status and the formation of an anti-tumor immune milieu. Thus, the MDS provides an accessible biological window into the dynamic changes in host systemic metabolic and inflammatory responses for predicting pCR. Moreover, patient nutritional status and body composition (e.g., sarcopenia) have been shown to modulate tumor response to therapy by influencing metabolic and inflammatory pathways. Although systematic records of nutritional interventions were unavailable in our retrospective data to quantify its independent effect, this potential confounder suggests that future prospective studies should integrate standardized nutritional assessment and intervention to further elucidate the complex relationships among the metabolic dynamic score, nutritional status, and treatment outcomes. Other metabolic indicators such as TG, glucose, and LDH have certain predictive value for rectal cancer progression (49, 50), but in our study, these indicators showed no statistical significance pre- or post-NCRT (P>0.05) and, therefore, were not included in the scoring system.

Compared to complex and costly modalities like high-resolution MRI radiomics (e.g., mrTRG), circulating tumor DNA (ctDNA) analysis, or PET/CT metabolic parameters (18), the strength of the MDS lies in its basis in routine, low-cost blood tests. Our goal is not to propose the MDS as a replacement for these established or emerging standards, but to explore its potential as a widely accessible and convenient supplementary source of information. Other metabolic indicators (TG, LDH, glucose) showed no significant dynamic association with pCR in our cohort and were not included.

To date, ML has been extensively used in the medical sector (51). For example, for predicting treatment responses in cancer patients, it has proven more effective than traditional prediction models (20, 52). Our previous work combining inflammatory and nutritional indicators with ML to predict pCR rates in patients with LARC also showed good predictive performance (53). Zhang et al. used three ML methods to study pCR rates among patients with LARC receiving NCRT (54). However, their study had relative limitations in ML model selection. In the current study, we employed 10 ML prediction models to minimize this limitation. Among the 10 constructed ML models, XGBoost demonstrated superior predictive performance (AUROC = 0.93, AP = 0.732) to those of the other nine models.

The interpretability of ML prediction models was shown using SHAP values. These models assessed and quantified the significance of each variable and ultimately presented it in the form of SHAP diagrams. Using SHAP values, we optimized the model to help clinicians better understand how the model makes decisions and facilitate personalized medical decision-making for patients (55–57). In this study, we analyzed variables related to pCR and concluded from SHAP diagrams that cN stage, tumor size, cT stage, and the new MDS were all significantly correlated with pCR.

In the context of multidisciplinary team (MDT) management for locally advanced rectal cancer (LARC), our model provides objective, data-driven estimates of pathological complete response (pCR) probability at the post-neoadjuvant chemoradiotherapy (NCRT) decision point, using routine blood test results and basic clinical variables. Based on these estimates, patients can be clearly stratified: those with a high predicted probability may be considered for a watch-and-wait strategy or organ-preserving approaches to optimize quality of life, whereas those with a low probability warrant early surgical intervention or intensified adjuvant therapy to avoid under treatment. Although the training cohort (2010–2017) was based on conventional chemoradiotherapy, whereas TNT and short-course radiotherapy are now standard, the model retains value in contemporary practice. Its pretreatment baseline variables enable stratification before strategy selection, helping identify patients likely to benefit from TNT intensification or watch-and-wait. However, different regimens may affect metabolic dynamics and cut-off values, necessitating validation in prospective cohorts on current TNT protocols. The online platform features a straightforward interface and requires no additional software installation, allowing rapid access during MDT meetings. This integration facilitates the combination of objective predictive outputs with clinical judgment, thereby promoting more precise and individualized therapeutic decisions.

As our study used a retrospective research method, some selection bias was inevitable. Additionally, variations in chemoradiotherapy regimens and treatment durations among patients with LARC may have relative impacts on the validity of our predictive model. Another limitation is that, while we recognize nutritional status may modulate treatment response by influencing the metabolic indicators central to this study as well as systemic inflammation, we were unable to incorporate it as a covariate for precise adjustment due to the lack of uniform, detailed quantitative nutritional intake data or systematic body composition analysis for all patients in this retrospective setting. Furthermore, although we performed an external validation using an independent cohort from a second center, both centers are located within the same province, which may limit the generalizability of our model to broader geographic regions and diverse ethnic populations. We acknowledge this as a key constraint on the true external validity of our findings. To address this, we have made our predictive platform openly accessible online to facilitate easy application in different clinical settings, and we are actively pursuing collaborations with other domestic and international institutions for prospective multi-center validations. In the future, we aim to incorporate more clinical factors to improve our ML prediction model, thereby enhancing its accuracy and providing better decision-making support for clinicians.

As our study used a retrospective research method, some selection bias was inevitable. The neoadjuvant chemotherapy regimens in this study were heterogeneous, including CapeOX, capecitabine monotherapy, and FOLFOX. Due to the limited sample size within each subgroup, we did not incorporate regimen type into the model to avoid overfitting, as our model is intended for pre-treatment baseline risk stratification rather than for comparing efficacy across different regimens. Consequently, variations in chemoradiotherapy regimens and treatment durations, along with the lack of regimen-stratified analysis due to limited subgroup sample sizes, may have relative impacts on the generalizability of our predictive model. The applicability of this model to contemporary TNT or short-course radiotherapy protocols warrants further validation in prospective cohorts with larger, uniformly treated populations. Another limitation is that, while we recognize nutritional status may modulate treatment response by influencing the metabolic indicators central to this study as well as systemic inflammation, we were unable to incorporate it as a covariate for precise adjustment due to the lack of uniform, detailed quantitative nutritional intake data or systematic body composition analysis for all patients in this retrospective setting. Furthermore, although we performed an external validation using an independent cohort from a second center, both centers are located within the same province in China, which substantially limits the generalizability of our model to broader geographic regions and diverse ethnic populations, as patients from this region share similar genetic backgrounds, lifestyles, and healthcare practices. We acknowledge this as the most critical constraint on the true external validity of our findings. To address this, we have made our predictive platform openly accessible online to facilitate easy application in different clinical settings, and we are actively pursuing collaborations with other domestic and international institutions for prospective multi-center validations across diverse populations. In the future, we aim to incorporate more clinical factors to improve our ML prediction model, thereby enhancing its accuracy and providing better decision-making support for clinicians.

## 5 Conclusion

Here, we collected and analyzed more readily accessible biochemical metabolic indicators pre- and post-NCRT to predict patients’ likelihoods of achieving a pCR and established MDS, a novel predictive factor. We proved that this factor independently predicts pCR. We then combined this predictive factor with patients’ clinical characteristics and constructed predictive models using ML techniques. Through model evaluation, we identified the optimal predictive model. The constructed ML prediction model demonstrates promising performance in predicting pCR outcomes in our cohort. With external validation in broader populations, it has the potential to serve as a valuable reference for clinicians. For now, it should be viewed as an exploratory tool rather than a definitive guide for treatment decisions.

## Data Availability

The raw data supporting the conclusions of this article will be made available by the authors, without undue reservation.
