# Supplementary material for: Metabolic dynamic score and machine learning: a novel approach to predicting pathological complete response in rectal cancer after neoadjuvant chemoradiotherapy
Source: Front Oncol. 2026 Aug 7;16:1910909. doi: 10.3389/fonc.2026.1910909 (PMC13493285; doi:10.3389/fonc.2026.1910909)
Supplement: Supplementary file 1 [file Table1.docx]

| Supplementary Table S1. Baseline characteristics of biochemical markers | | | | |
| --- | --- | --- | --- | --- |
| **Biomarker** | **Training cohort (n=650)** | **Internal validation (n=196)** | **External validation (n=454)** | **P-value** |
| pre-TBIL | 11.22±5.14 10.10 (7.70;13.40) [2.60-35.20, n=650] | 10.37±4.45 9.70 (7.38;12.50) [3.50-32.30, n=196] | 10.87±4.92 9.80 (7.60;12.80) [3.30-33.30, n=454] | 0.179 |
| pre-DBIL | 3.52±2.78 2.90 (2.23;4.30) [0.80-56.00, n=650] | 3.02±3.96 2.60 (2.00;3.30) [0.80-56.00, n=196] | 3.18±2.92 2.80 (2.10;3.60) [0.80-56.00, n=454] | <0.001 |
| pre-IDIL | 10.45±63.90 7.10 (5.10;9.40) [1.50-1631.00, n=650] | 7.59±3.53 7.00 (5.20;9.33) [2.40-26.60, n=196] | 11.36±76.28 7.00 (5.23;9.38) [1.80-1631.00, n=454] | 0.933 |
| pre-GGT | 30.47±28.00 23.00 (16.00;36.00) [5.00-450.00, n=650] | 26.14±33.02 21.00 (15.00;29.50) [6.00-450.00, n=196] | 26.98±26.57 21.00 (15.00;31.75) [5.00-450.00, n=454] | 0.006 |
| pre-ALP | 73.48±19.86 72.00 (61.00;85.00) [7.00-188.00, n=650] | 74.30±21.01 73.50 (63.00;85.00) [13.00-188.00, n=196] | 72.37±20.14 71.00 (59.00;83.00) [7.00-188.00, n=454] | 0.325 |
| pre-TC | 4.85±1.13 4.79 (4.23;5.39) [0.35-14.00, n=650] | 5.01±1.08 4.91 (4.38;5.52) [2.74-13.11, n=196] | 4.88±1.10 4.81 (4.26;5.39) [0.35-14.00, n=454] | 0.206 |
| pre-LDL | 3.24±0.88 3.21 (2.66;3.73) [0.99-11.15, n=650] | 3.32±0.98 3.25 (2.78;3.80) [0.99-11.15, n=196] | 3.27±0.89 3.22 (2.69;3.75) [0.99-11.15, n=454] | 0.672 |
| pre-HDL | 1.32±0.35  1.28(1.10;1.50)  [0.50-3.20, n=650] | 1.35±0.37  1.30(1.12;1.52)  [0.55-3.00, n=196] | 1.33±0.36  1.29(1.11;1.51)  [0.50-3.20, n=454] | 0.421 |
| pre-TG | 1.45±0.85  1.30(0.90;1.80)  [0.35-8.00, n=650] | 1.53±0.87  1.36(0.95;1.88)  [0.40-7.50, n=196] | 1.46±0.86  1.31(0.91;1.82)  [0.35-8.00, n=454] | 0.385 |
| pre-ApoA | 1.37±0.28  1.35(1.20;1.52)  [0.60-2.80, n=650] | 1.38±0.29  1.36(1.21;1.53)  [0.65-2.60, n=196] | 1.38±0.27  1.36(1.21;1.52)  [0.60-2.80, n=454] | 0.912 |
| pre-ApoB | 0.87±0.24  0.85(0.73;0.99)  [0.25-2.00, n=650] | 0.88±0.25  0.86(0.74;1.00)  [0.30-1.90, n=196] | 0.87±0.23  0.85(0.73;0.99)  [0.25-2.00, n=454] | 0.873 |
| post-TBIL | 12.27±5.58 11.05 (8.80;14.40) [1.50-52.90, n=650] | 11.31±4.80 10.20 (8.10;13.03) [2.90-32.90, n=196] | 11.82±5.18 10.95 (8.60;13.80) [1.50-52.90, n=454] | 0.051 |
| post-DBIL | 3.94±2.31 3.30 (2.50;4.70) [0.60-23.90, n=650] | 3.09±1.19 2.80 (2.30;3.60) [0.90-8.70, n=196] | 3.41±1.63 3.00 (2.40;3.90) [0.60-18.70, n=454] | <0.001 |
| post-IDIL | 8.54±5.02 7.70 (5.70;10.00) [0.50-77.00, n=650] | 8.21±3.81 7.40 (5.60;9.70) [1.90-26.00, n=196] | 8.65±5.23 7.90 (5.90;10.00) [0.50-77.00, n=454] | 0.439 |
| post-GGT | 33.89±27.63 25.00 (18.00;39.00) [3.00-294.00, n=650] | 29.81±20.46 24.00 (18.00;35.00) [3.00-154.00, n=196] | 32.02±24.49 25.00 (18.00;37.00) [3.00-220.00, n=454] | 0.378 |
| post-ALP | 78.19±25.34 75.00 (63.00;90.00) [0.00-212.00, n=650] | 78.43±21.07 77.00 (66.75;90.00) [7.90-152.00, n=196] | 76.46±23.27 73.00 (62.00;88.00) [0.00-212.00, n=454] | 0.264 |
| post-TC | 5.09±1.08 5.00 (4.36;5.68) [1.04-12.60, n=650] | 5.21±1.09 5.12 (4.45;5.84) [3.00-10.98, n=196] | 5.20±1.12 5.09 (4.41;5.79) [2.11-12.60, n=454] | 0.245 |
| post-LDL | 4.03±15.76 3.30 (2.74;3.90) [1.27-404.00, n=650] | 3.53±1.72 3.32 (2.83;3.89) [1.27-23.20, n=196] | 4.40±18.84 3.36 (2.82;4.00) [1.27-404.00, n=454] | 0.291 |
| post-GLU | 5.74±6.87 5.16 (4.78;5.79) [3.64-178.00, n=650] | 6.21±12.38 5.10 (4.80;5.50) [4.00-178.00, n=196] | 5.74±8.20 5.04 (4.75;5.53) [3.64-178.00, n=454] | 0.032 |
| post-HDL | 1.40±0.38  1.36(1.18;1.58)  [0.60-3.50, n=650] | 1.43±0.39  1.38(1.20;1.60)  [0.65-3.30, n=196] | 1.41±0.37  1.37(1.19;1.59)  [0.60-3.50, n=454] | 0.587 |
| post-TG | 1.55±0.89  1.38(0.98;1.89)  [0.40-9.50, n=650] | 1.61±0.91  1.44(1.02;1.96)  [0.45-8.20, n=196] | 1.58±0.87  1.41(1.00;1.92)  [0.40-9.50, n=454] | 0.450 |
| post-ApoA | 1.32±0.25  1.30(1.16;1.45)  [0.70-2.50, n=650] | 1.35±0.26  1.33(1.19;1.48)  [0.75-2.30, n=196] | 1.33±0.24  1.31(1.18;1.46)  [0.70-2.50, n=454] | 0.320 |
| post-ApoB | 0.85±0.22  0.83(0.71;0.97)  [0.30-1.80, n=650] | 0.87±0.23  0.85(0.73;0.99)  [0.35-1.70, n=196] | 0.86±0.21  0.84(0.72;0.98)  [0.30-1.80, n=454] | 0.510 |
| Δ-TBIL | +1.05±7.59 +0.95 (+1.10;+1.00) [-1.10-+17.70, n=650] | +0.94±6.55 +0.50 (+0.72;+0.53) [-0.60-+0.60, n=196] | +0.95±7.14 +1.15 (+1.00;+1.00) [-1.80-+19.60, n=454] | 0.011 |
| Δ-DBIL | +0.42±3.61 +0.40 (+0.27;+0.40) [-0.20--32.10, n=650] | +0.07±4.13 +0.20 (+0.30;+0.30) [+0.10--47.30, n=196] | +0.23±3.34 +0.20 (+0.30;+0.30) [-0.20--37.30, n=454] | 0.073 |
| Δ-IDIL | -1.91±64.10 +0.60 (+0.60;+0.60) [-1.00--1554.00, n=650] | +0.62±5.19 +0.40 (+0.40;+0.37) [-0.50--0.60, n=196] | -2.71±76.46 +0.90 (+0.67;+0.62) [-1.30--1554.00, n=454] | 0.281 |
| Δ-GGT | +3.42±39.34 +2.00 (+2.00;+3.00) [-2.00--156.00, n=650] | +3.67±38.84 +3.00 (+3.00;+5.50) [-3.00--296.00, n=196] | +5.04±36.13 +4.00 (+3.00;+5.25) [-2.00--230.00, n=454] | 0.698 |
| Δ-ALP | +4.71±32.20 +3.00 (+2.00;+5.00) [-7.00-+24.00, n=650] | +4.13±29.76 +3.50 (+3.75;+5.00) [-5.10--36.00, n=196] | +4.09±30.78 +2.00 (+3.00;+5.00) [-7.00-+24.00, n=454] | 0.378 |
| Δ-TC | +0.24±1.56 +0.21 (+0.13;+0.29) [+0.69--1.40, n=650] | +0.20±1.53 +0.21 (+0.07;+0.32) [+0.26--2.13, n=196] | +0.32±1.57 +0.28 (+0.15;+0.40) [+1.76--1.40, n=454] | 0.370 |
| Δ-LDL | +0.79±15.78 +0.09 (+0.08;+0.17) [+0.28-+392.85, n=650] | +0.21±1.98 +0.07 (+0.05;+0.09) [+0.28-+12.05, n=196] | +1.13±18.86 +0.14 (+0.13;+0.25) [+0.28-+392.85, n=454] | 0.708 |
| Δ-HDL | +0.08±0.43  +0.07(+0.05;+0.10)  [-1.20-+1.80, n=650] | +0.08±0.40 +0.07(+0.05;+0.09)  [-0.90-+1.50, n=196] | +0.08±0.42 +0.07(+0.05;+0.10)  [-1.20-+1.80, n=454] | 0.992 |
| Δ-TG | +0.10±0.75  +0.05(-0.08;+0.18)  [-2.50-+3.50, n=650] | +0.08±0.72  +0.04(-0.10;+0.16)  [-2.00-+3.00, n=196] | +0.12±0.77  +0.07(-0.07;+0.20)  [-2.50-+3.50, n=454] | 0.750 |
| Δ-ApoA | -0.05±0.30  -0.03(-0.10;+0.05)  [-1.20-+0.80, n=650] | -0.03±0.28  -0.02(-0.08;+0.06)  [-1.00-+0.70, n=196] | -0.06±0.31  -0.04(-0.11;+0.04)  [-1.20-+0.80, n=454] | 0.420 |
| Δ-ApoB | -0.02±0.25  -0.01(-0.07;+0.06)  [-0.90-+0.70, n=650] | -0.01±0.24  0.00(-0.06;+0.07)  [-0.80-+0.60, n=196] | -0.03±0.26  -0.02(-0.08;+0.05)  [-0.90-+0.70, n=454] | 0.610 |
| Values are presented as Mean±SD, Median (Q1;Q3) [Range, n=sample size] | | | | |
