# Supplementary material for: Metabolic dynamic score and machine learning: a novel approach to predicting pathological complete response in rectal cancer after neoadjuvant chemoradiotherapy
Source: Front Oncol. 2026 Aug 7;16:1910909. doi: 10.3389/fonc.2026.1910909 (PMC13493285; doi:10.3389/fonc.2026.1910909)
Supplement: Supplementary file 2 [file Table2.docx]

| Supplementary Table S2. Performance Evaluation and Comparison of the XGBoost Model Across Sex Subgroups in the Test Set | | | |  |
| --- | --- | --- | --- | --- |
| **Performance Metric** | **Male (n=351)** | **Female (n=299)** | **P-value (Male vs. Female)** | |
| AUC | 0.93 (0.90-0.96) | 0.92 (0.89-0.95) | 0.65 (DeLong's test) | |
| Precision | 0.88 (0.85-0.91) | 0.91 (0.88-0.94) | 0.45 (Chi-square test) | |
| Recall | 0.89 (0.86-0.92) | 0.90 (0.87-0.93) | 0.80 (Chi-square test) | |
| F1-score | 0.88 (0.85-0.91) | 0.90 (0.87-0.93) | 0.50 (Chi-square test) | |
| Accuracy | 0.89 (0.86-0.92) | 0.90 (0.87-0.93) | 0.70 (Chi-square test) | |
